# Transcriptome analysis of the white pine blister rust pathogen *Cronartium ribicola*: *de novo* assembly, expression profiling, and identification of candidate effectors

**DOI:** 10.1186/s12864-015-1861-1

**Published:** 2015-09-04

**Authors:** Jun-Jun Liu, Rona N. Sturrock, Richard A. Sniezko, Holly Williams, Ross Benton, Arezoo Zamany

**Affiliations:** Pacific Forestry Centre, Canadian Forest Service, Natural Resources Canada, 506 West Burnside Road, Victoria, BC V8Z 1M5 Canada; USDA Forest Service, Dorena Genetic Resource Center, 34963 Shoreview Road, Cottage Grove, OR 97424 USA

**Keywords:** *Cronartium ribicola*, Effector, Pathogenicity, RNA-seq, Transcriptome profiling

## Abstract

**Background:**

The fungus *Cronartium ribicola* (Cri) is an economically and ecologically important forest pathogen that causes white pine blister rust (WPBR) disease on five-needle pines. To cause stem cankers and kill white pine trees the fungus elaborates a life cycle with five stages of spore development on five-needle pines and the alternate host *Ribes* plants. To increase our understanding of molecular WP-BR interactions, here we report genome-wide transcriptional profile analysis of *C. ribicola* using RNA-seq.

**Results:**

cDNA libraries were constructed from aeciospore, urediniospore, and western white pine (*Pinus monticola*) tissues post Cri infection. Over 200 million RNA-seq 100-bp paired-end (PE) reads from rust fungal spores were *de novo* assembled and a reference transcriptome was generated with 17,880 transcripts that were expressed from 13,629 unigenes. A total of 734 unique proteins were predicted as a part of the Cri secretome from complete open reading frames (ORFs), and 41 % of them were *Cronartium*-specific. This study further identified a repertoire of candidate effectors and other pathogenicity determinants. Differentially expressed genes (DEGs) were identified to gain an understanding of molecular events important during the WPBR fungus life cycle by comparing Cri transcriptomes at different infection stages. Large-scale changes of *in planta* gene expression profiles were observed, revealing that multiple fungal biosynthetic pathways were enhanced during mycelium growth inside infected pine stem tissues. Conversely, many fungal genes that were up-regulated at the urediniospore stage appeared to be signalling components and transporters. The secreted fungal protein genes that were up-regulated in pine needle tissues during early infection were primarily associated with cell wall modifications, possibly to mask the rust pathogen from plant defenses.

**Conclusion:**

This comprehensive transcriptome profiling substantially improves our current understanding of molecular WP-BR interactions. The repertoire of candidate effectors and other putative pathogenicity determinants identified here are valuable for future functional analysis of Cri virulence and pathogenicity.

**Electronic supplementary material:**

The online version of this article (doi:10.1186/s12864-015-1861-1) contains supplementary material, which is available to authorized users.

## Background

White pine blister rust (WPBR) caused by *Cronartium ribicola* (Cri) is a devastating fungal disease of five-needle pines (subgenus Strobus) around world. Since the early 20th century, when it was accidently introduced into North America, WPBR has spread over the continent where native five-needle pine species were distributed, with severe ecological and economic damages. WPBR has decreased western white pine (WWP, *Pinus monticola*) populations up to 90 %, and also seriously disturbed forest ecosystems of other native five-needle pines. Breeding programs for selection of genetic resistance to WPBR have been undertaken in sugar pine (*P. lambertiana*), WWP (*P. monticola*), eastern white pine (*P. strobus*), whitebark pine (*P. albicaulis*), limber pine (*P. flexilis*) and others with significant progress [1]. Both major gene resistance (MGR) and quantitative disease resistance have been discovered and utilized in the five-needle pine breeding and conservation programs. MGR has been reported in four five-needle pine species: *P. lambertiana*, *P. monticola*, *P. strobiformis*, and *P. flexilis* [2]; and it is characterized by hypersensitive response (HR)-like reactions in the pine needles infected by *C. ribicola* [3]. This reaction is characterized by a rapid induction of host cell death and subsequent localized tissue necrosis, which prevents spread of the rust mycelium to vascular stem tissue. However, Cri virulent races (*vcr1* and *vcr2*) have arisen and the corresponding breakdown of MGR have been documented in regions where resistant *P. lambertiana* and *P. monticola* are planted [4]. Therefore, WPBR is still the major constraint to re-plantation of five-needle pines for the forest industry and restoration of ecosystems in western North America.

**Fig. 1:**
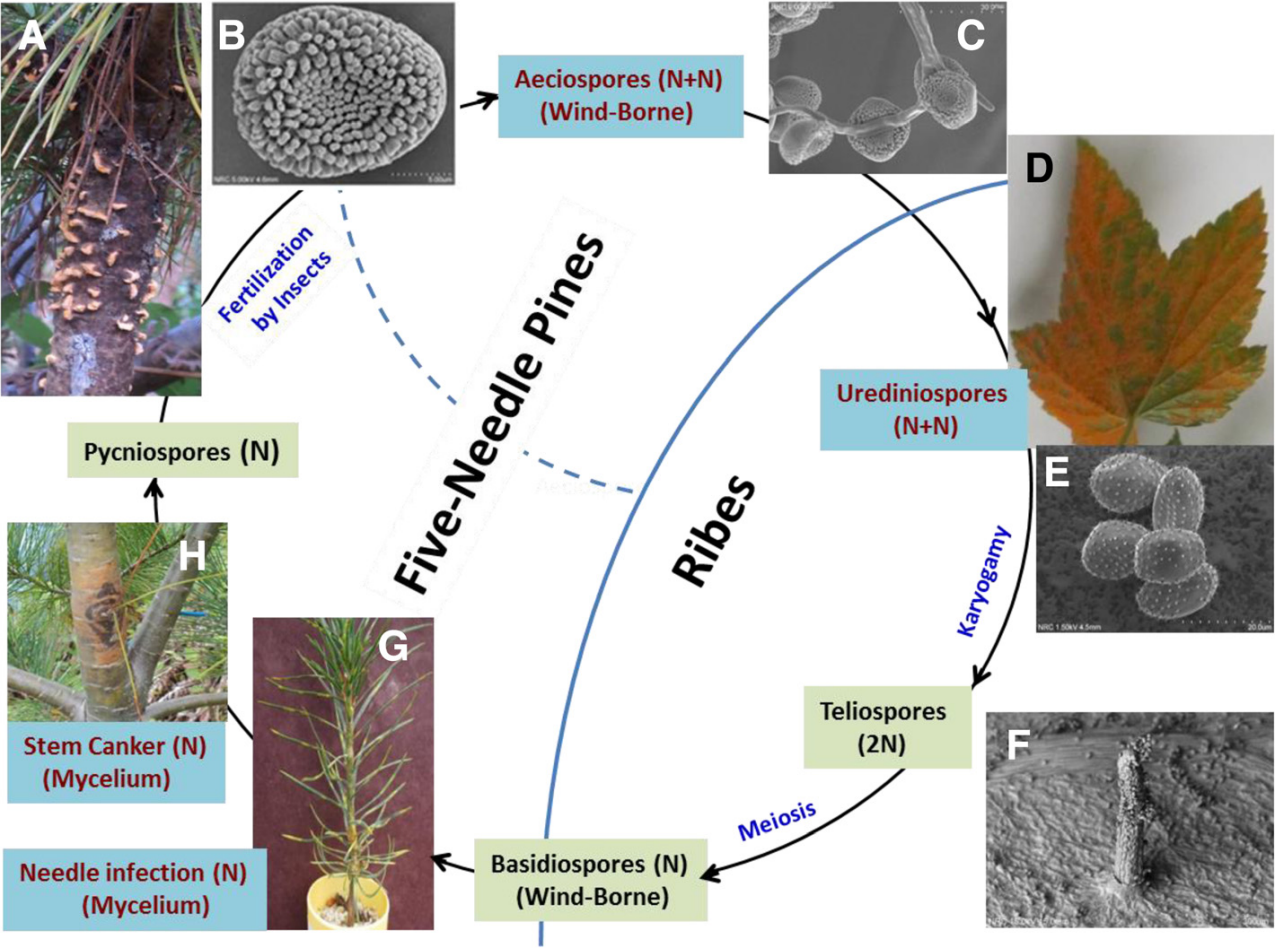
*Cronartium ribicola* life cycle with five stages of spore development. **a** Blisters on the infected white pine stem; **b** Aeciospore; **c** Aeciospore germination; **d** Rust fungus growth on an infected *Ribes* leaf; **e** Urediniospores; **f** Telia on *Ribes* leaf; **g** One-year-old susceptible seedling ~6 months (March) post needle infection by basidiospores; **h** A typical canker on western white pine stem ~20 months post infection on needles. The four rust development stages sampled for comparative analysis of rust fungal transcriptomes are indicated by red letters

*C. ribicola* is an obligate biotrophic fungus and requires an alternate host plant (mainly *Ribes* species) for completion of its life cycle (Fig. 1) [5]. In spring (or summer for high elevation species like whitebark pine) aeciospores are released from stem cankers of susceptible five-needle pines and dispersed by air onto *Ribes* plants. Aeciospores germinate on *Ribes* leaves to initiate the asexual stage of infection, which involves mycelium growth in *Ribes* leaf tissue, sporulation to produce urediniospores, and repeated infection of nearby *Ribes* by urediniospores throughout the summer season. In late summer or early fall, telia begin to grow and produce rows of teliospores. As the weather becomes wet and cooler, teliospores germinate in place and produce basidia, where basidiospores are developed, dispersed via air movement and subsequently to infect pine host. The germinated basidiospore enters pine needles through stomata, and hyphae then grow along vascular tissues into the branch and stem. The mycelium continues to spread in the bark tissues of susceptible five-needle pines, resulting in a swollen canker in the next spring or summer.

During the initial infection stage by basidiospores, a typical haustorium-pine cell interface was observed in the WPBR pathosystem [6]. Haustorially expressed secreted proteins (HESPs), including effectors, are proposed to play key roles in manipulating the immune responses of host cells [7]. Effectors are microbial and pest secreted molecules that alter host-cell processes or structures to generally promote their own lifestyle. Effector functions are as diverse as suppressing immune responses to enhancing access to nutrients [8]. There are at least four avirulence (Avr) effectors (avcr1 to avcr4) and two virulence effectors (vcr1 and vcr2) in *C. ribicola* [2, 4]. However, the molecular identities of these Cri effectors are unknown, and as is how they reprogram biological processes to facilitate rust pathogen growth and to mitigate host defenses in five-needle pines. Virulence effectors overcome plant immunity by modifying host metabolism to support pathogen growth and spread, which is termed as effector-triggered susceptibility (ETS). Avr effectors are recognized by specific host receptors encoded by plant resistance (R) genes to activate a host defense response termed as effector-triggered immunity (ETI) [8]. Some Avr effectors translocate into plant cells where their activity is recognized for re-programming plant transcription, metabolism, and defense during pathogen infection [9]. The virulence effectors play a crucial role in virulent fungal races overcoming plant MGR by escaping ETI.

Next generation sequencing technologies and bioinformatics have rapidly advanced in recent years. Genome sequences of four rust fungal species have been reported: *Melampsora larici-populina* (Mpl), *M. lini* (Mli), *Puccinia graminis f.sp. tritici* (Pgt), and *P. striiformis f.sp. tritici* (Pst) [10–13]. Based on bioinformatic analyses and gene expression studies of the infected host tissues or haustoria, 8 to12% of the predicted proteomes of these rust fungi corresponds to secreted proteins as candidate effectors. RNA-seq has become an instrumental assay for the analysis of multiple aspects of fungal transcriptomes, such as transcriptional profiling [14–17], identification of putative virulence genes [18], secretome analysis [19, 20], gene models and alternative transcript splicing [21], and structural gene annotations [22]. These genomic studies provide valuable research resources and tools in understanding the dynamics of plant-rust pathogen interactions.

To provide genomic insight into *in planta* transcriptome profiles of the WPBR pathosystem, we performed *de novo* sequencing of the Cri transcriptome using RNA-seq analysis during infection of WWP and the alternate host, *Ribes*. We assessed Cri transcriptomes and predicted 734 secreted proteins. Global gene expression profiling allowed us to characterize transcript expression of genes that have putative key roles during biotrophic infection and mycelial growth inside host tissues. In particular, identification of candidate effectors provides novel insights into molecular WP-BR interactions, which until now have been poorly understood. This first large-scale genomics resource of the WPBR pathosystem provides the relevant sequences and gene regulation information for gene discovery, functional and population genomics, comparative analyses, and future efforts to annotate its genome. This resource permits the study of this rust fungus at the genome level as well as providing data for researchers working with other *Cronartium* species.

## Results

### *De novo* assembly of Cri transcriptomes

Fungal samples at Cri life cycle stages of aeciospore, urediniospore, and mycelium growth in infected WWP needles and cankered stems were used for RNA-seq analysis (Fig. 1). We used aeciospore- and urediniospore-derived RNA-seq reads (>200 million 100-bp PE reads from six cDNA libraries) in *de novo* assembly for construction of a Cri reference transcriptome (see method section), which resulted in 17,880 transcripts expressed from 13,629 unigenes for further analysis. This Cri reference transcriptome had a total length of 21.7Mb, N50 of 2,084-bp, and average length of 1,213-bp (Additional file 1: Table S1). TransDecoder predicted the Cri reference proteome with a total of 17,264 putative proteins with minimum length of 50 amino acids, 9,228 (53.5 % of the total) open reading frames (ORFs) were complete sequences (Additional file 1: Table S2). The Cri reference transcriptome (i.e., 17,880 contigs >200 nt) has been deposited at GenBank as a transcriptome shotgun assembly (TSA) under accession GBSG00000000.

RNA-seq reads from infected pine stem tissues were used for *de novo* assembly of dual transcriptomes of *C. ribicola* and *P. monticola*. By alignment analysis of dual transcriptomes with the Cri reference transcriptome generated here and *P. monticola* stem reference transcriptome [23], a total of 23,671 sequences were separated as the Cri associated transcripts that were *in planta* expressed in the infected stems (Additional file 1: Table S1).

### Annotation of the Cri reference transcriptome

Compared with Cri cDNA sequences (AF232039 and AF353616) available from Genbank, *de novo* assembled transcripts showed 100 % identity to them. Over 94 % of the core eukaryotic gene (CEG) set, which contains 2,748 CEG variants from six eukaryotic genomes [24], matched peptides of the Cri reference proteome, with an average of 89 % of the protein length aligned (STD: ± 17 %; BLASTp e-value < e^-5^). BLASTp analysis of the Cri reference proteome against Mlp-, Mli-, Pst-, and Pgt-proteomes revealed that 51–59 % of Cri proteins were conserved between Cri and other rust fungi (Additional file 1: Table S3). BLASTp with reciprocal best hits (RBH) identified 4,986 orthologs (36.6 % of total Cri genes) between Cri and Mlp. Similarly, OrthoMCL analysis assigned putative Cri proteins into 4,104 ortholog groups. The number of rust fungal orthologs from *C. ribicola* is very close to the reports on other rust pathogens [11, 12], indicating that the core genes are well covered in the Cri reference transcriptome.

Gene annotation revealed that about 60 % of total transcripts (10,667 out of 17,880) showed homology hits (E values < 10^−5^) in a search against the NCBI nr database, and 43 % of them (7,676) were assigned at least one gene ontology (GO) term. A BLASTx top-hit species distribution of gene annotations showed highest homology to Mlp (63 %), followed by Pgt (15 %), *Baudoinia compniacensis* (1 %), and *Dothistroma septosporum* (0.9 %) (Additional file 2: Figure S1). This result suggests that Cri may be closest to Mlp at the transcriptome level based on the databases reported so far.

After assigning Cri transcripts to the categories of cellular processes, molecular functions, and cellular components (Additional file 3: Figure S2), GO enzyme code mapping further identified 372 unique enzyme codes for 2,429 Cri proteins, which were involved in 113 metabolic pathways. The dbCAN v3.0 HMM-based CAZy annotation [25] identified 310 Cri CAZy-like proteins with assignment to 89 CAZy families. Of all annotated CAZymes, 41.8 % were grouped into 35 families of glycosyl hydrolases (GH), 31.6 % to 25 families of glycosyl transferases (GT), 12.6 % to seven families of carbohydrate esterases (CE, not including CE10), 3.5 % to four polysaccharide lyase (PL) families, 4.4 % to 11 families of carbohydrate binding modules (CBM), and 6.0 % to seven families of redox enzymes that act in conjunction with CAZymes (AA) (Additional file 1: Table S4). The top GH families are GH5 (cellulases/hemicellulases), GH18 (chitinases), GH16 (β-1,3-glucanas), and GH47 (α-1,2-mannosidases), containing 18, 13, 12, and 10 proteins respectively, consistent with recent findings in Pst, Pgt, and Mlp [11, 26]. The three most abundant CE families were CE4 (chitin deacetylases), CE1 (acetyl xylan esterases), and CE8 (pectin methylesterases). Among the GT group, GT2 (cellulose/chitin synthase), GT32 (mannosyltransferases), and GT90 (xylosyltransferases) were extensively expanded with more expressed genes than others (Additional file 1: Table S4). A BLASTp search against the Transporter Classification Database [27] identified 1,195 Cri proteins (7 % of the proteome) related to transporter activity and they were assigned to 116 transporter families (Additional file 1: Table S5). The most abundant transporter family was the major facilitator superfamily (MFS), functioning for the uptake of sugars, oligosaccharides, amino acids, metabolites, and others.

### Identification of secreted proteins and candidate effectors

**Fig. 2:**
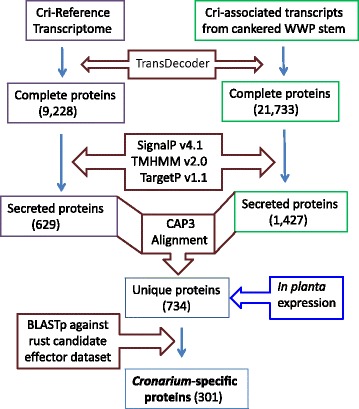
Workflow of secretome prediction using bioinformatics tools for the comprehensive characterization of proteins secreted at spore development stages or during the *in-planta* mycelium growth stage in infected stems of western white pine

Because secreted proteins have potential roles in plant-microbe interactions, we analyzed the Cri secretome based on transcript assemblies (Fig. 2). Secreted proteins were encoded by 629 transcripts in the Cri reference transcriptome that originated from rust fungal spores and 1,427 transcripts in the Cri-associated transcripts that originated from cankered WWP stems. Following CAP3 alignment of the complete ORFs of these secreted protein-encoding transcripts, a total of 734 unique proteins were identified as secreted proteins. A BLASTp search against a dataset of candidate effectors with 23,516 proteins from the other four rust pathogens [13] showed that 301 (41 % of the total) Cri secreted proteins were *Cronartium*-specific, as they showed no significant homology to candidate effectors reported on other rust fungi (E values ˃ e^−5^) (Additional file 1: Table S3). Because a large number of pathogenic effectors share features as secreted, small and cysteine-rich proteins (SSPs) that lack homology to proteins in other species, we examined the Cri secretome and manual curation found that it contained 466 proteins with lengths shorter than 300 amino acids and 155 of them had at least four cysteine residues in the mature proteins. As anticipated, most of these Cri SSPs (82 %) had no functional annotation as shown by BLAST2GO analysis.

Of annotated Cri secreted proteins, 85 belonged to 39 families of CAZymes (Additional file 1: Table S4), and 36 % of the Cri secretome contained distinct Pfam-A domains (Additional file 1: Table S6). Pfam domains abundant in secreted proteins included peptidases or proteases (such as Asp domain), CFEM (cysteine-rich fungal effector motif), Cu-oxidases, DPBB-1 (double psi beta-barrel structure), and GPI-anchored domains. Numerous fungal proteins with these domains have been reported with functional involvement in fungal pathogenicity. Other Pfam domains related to fungal pathogenicity, such as CAP (cysteine-rich secretory proteins), HSP (heat-shock protein), DnaJ/DnaJ-X (also known as HSP40), and thaumatin, were also identified in multiple Cri secreted proteins (Additional file 1: Table S6).

To recover additional secreted proteins among partial ORF sequences, the Cri reference proteome was used as a query in a BLASTp search against the Cri secreted protein dataset. To generate a more complete dataset of candidate effectors, Cri candidate effectors were found using a BLASTp search of the Cri reference proteome against candidate effectors from other rust fungis [13], then further filtered by a BLASTp search against the PHI database [28]. A total of 2,770 proteins (16 % of the reference proteome) were identified as Cri candidate effectors, of which 67.4 % (1868) contained a total of 907 distinct Pfam domains by Pfam annotation. Among these annotated domains, 152 domains were selected because the 881 Cri candidate effectors with these Pfam domains also showed significant homologies to the PHI proteins documented with phenotypic effects on virulence and pathogenicity, such as hypervirulence, loss of pathogenicity, reduced virulence, etc. (Additional file 1: Table S7). The largest clusters of protein families within the 881 candidate effectors were protein kinases (pkinase domain), small GTPases (Ras and Arf domains), CAZymes (GH18, GH72, and others), DEAD/DEAH box helicases, WD40 proteins, HSP70 and HSP90 proteins, and proteins with AAA domains and RNA recognition motifs (RRM).

### Analysis of transcriptome profiles

**Fig. 3:**
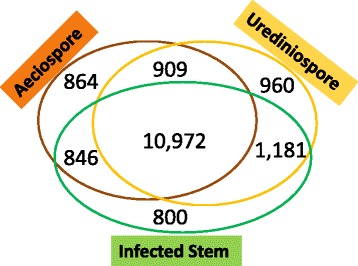
Venn diagram depicting transcripts expressed in *Cronartium ribicola* samples using RNA-seq. Numbers of contigs are indicated for the samples at the aeciospore stage collected from stem blisters of western white pine, at the urediniospore stage collected from infected *Ribes* leaves, and during *in-planta* mycelium growth from infected western white pine stems about 14 months after inoculation

**Fig. 4:**
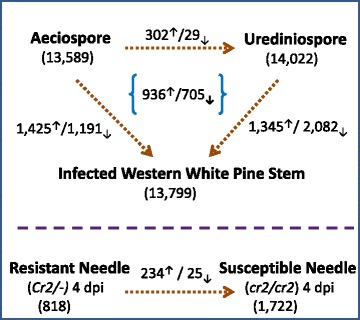
Numbers of differentially expressed transcripts by comparison of transcriptome profiles at the three life cycle stages of aeciospore, urediniospore, and *in-planta* mycelium growth in infected western white pine stem (*above*), or between resistant and susceptible needles at 4 dpi by rust basidiospores (*below*)

To investigate *in planta* rust fungal gene expression, trimmed RNA-seq reads were mapped to the Cri reference transcriptome. 86 % of the reads from aeciospores and 85 % of the reads from urediniospores were aligned to the transcripts while about 24 % and 6 % of the reads from infected stem and infected needle tissues respectively were derived from the Cri reference transcriptome (Table 1). Of the Cri reference transcriptome, 13,591 (76 %), 14,022 (78 %) and 13,799 (77 %) transcripts were detected at life cycle stages of aeciospore, urediniospore, and infected stem, respectively. In total, we detected 10,972 transcripts that were commonly expressed in all three types of samples. In contrast, only 864, 960, and 800 contigs were detected with exclusive expression in aeciospore, urediniospore, and infected stem respectively (Fig. 3). To further analyze the detailed infection regulatory program, RNA-seq analysis was performed for four pair-wise comparisons of transcriptome profiles: aeciospore vs. urediniospore, aeciospore vs. infected stem, urediniospore vs. infected stem, and susceptible needles vs. resistant needles infected by basidiospores at 4 days post infection (4 dpi). The full set of expression data (FPKM) for the Cri reference transcriptome is shown in Additional file 1: Table S8. Pearson correlation analysis showed good sample reproducibility for aeciospores (*R* = 0.98 ± 0.02), urediniospores (*R* = 0.94 ± 0.04), and infected stems (*R* = 0.87 ± 0.10), respectively. Transcripts with normalized fold change > ±2 (*p* <0.05 after adjustment with false discovery rate - FDR) in at least one paired comparison were considered differentially expressed genes (DEGs) with up- or down-regulation and their numbers are shown for each comparison in Fig. 4. As compared to both aeciospore and urediniospore, *in planta* mycelium growth inside infected stem tissues consistently triggered a higher number of up-regulated transcripts than down-regulated transcripts (936 vs. 705).

**Table 1:** Mapping of RNA-seq reads using *Cronartium* ribicola reference transcriptome (17,880 contigs)

Sample	cDNA Library	Mapped reads (n)	Total reads (n)	Mapped reads (%)	Expressed transcript/unigene (n)
Aeciospore	AB4	28,674,922	33,449,754	85.73 %	10,462/7,375
	AB3	41,873,710	48,161,514	86.94 %	10,886/7,622
	AB6	28,037,995	33,065,836	84.79 %	12,100/8,766
	Sub-total	98,586,627	114,677,104	85.97 %	13,589/9,884
Urediniospore	AA3	32,359,165	38,025,520	85.10 %	12,533/8,971
	AA8	18,171,226	20,924,558	86.84 %	11,497/8,149
	AAF	27,678,509	32,977,432	83.93 %	12,620/9,019
	Sub-total	78,208,900	91,927,510	85.08 %	14,022/10,191
Infected WWP stem	F3	13,325,832	59,693,634	22.32 %	12,292/8,966
	F5	18,449,393	75,975,818	24.28 %	12,325/8,977
	F8	23,381,136	89,845,810	26.02 %	12,582/9,175
	Sub-total	55,156,361	225,515,262	24.46 %	13,799/10,119
Infected WWP needle (4 dpi)	SUS (cr2/cr2)	6,033,538	116,335,954	5.19 %	1,722/1,542
	RES (Cr2/-)	11,018,625	141,232,768	7.80 %	818/770
	Sub-total	17,052,163	257,568,722	6.62 %	1,878/1,674

**Fig. 5:**
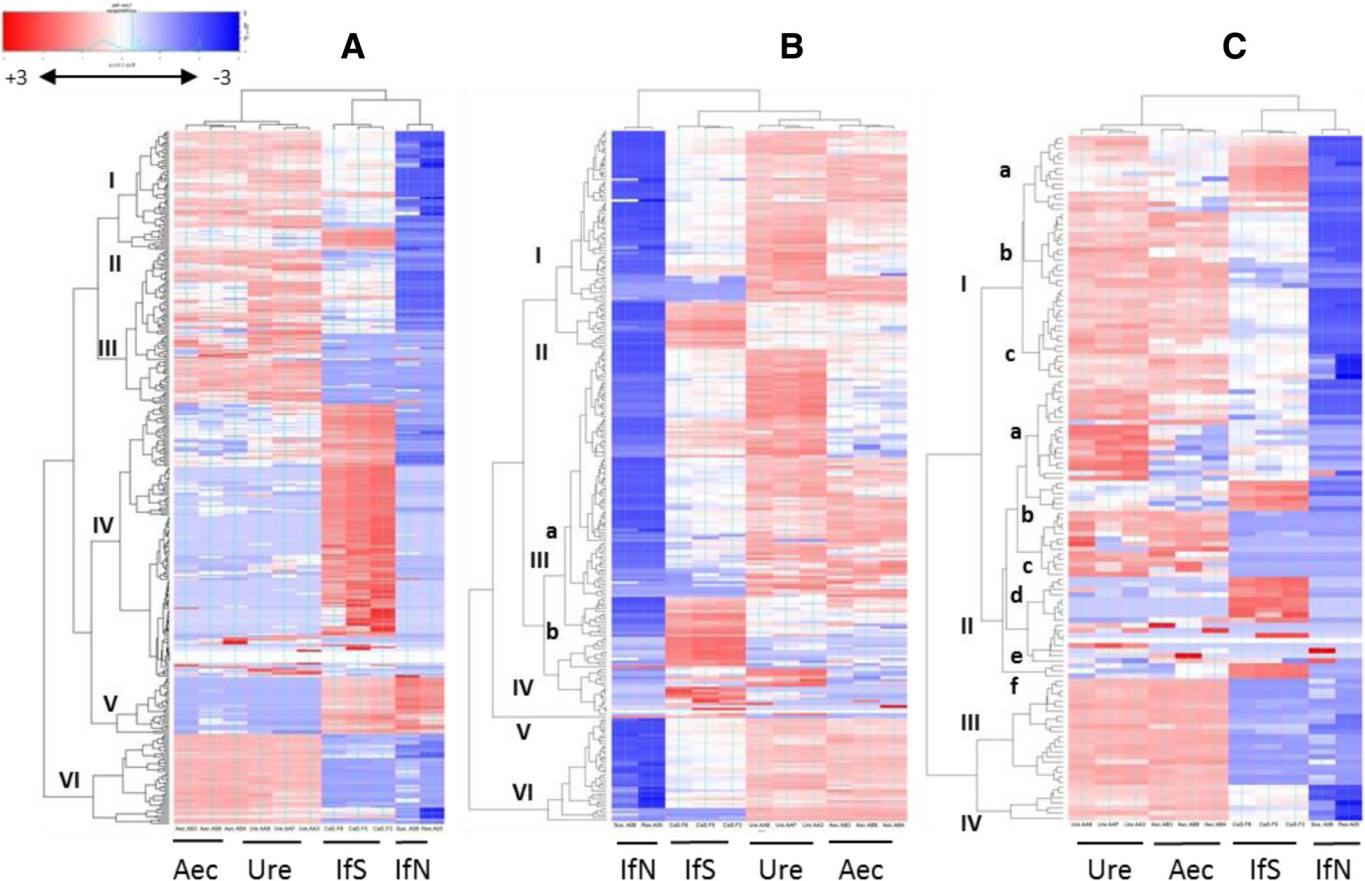
Heatmaps of transcript expression based on normalized data of expression values (FPKM) in three functional categories. Only differentially expressed genes (DEGs) with minimum fold change of two with *p* <0.05 after adjustment using false discovery rate (FDR) are shown in the heatmaps. Overrepresented (*red*) and underrepresented transcripts (*blue*) are shown as relative to the expression values measured across four stages of the rust life cycle, infected pine needle (IfN) at 4 dpi, infected pine stems (IfS), aeciospore (Aec), and urediniospore (Ure) in infected *Ribes* leaves. **a** Secreted proteins; **b** Candidate effectors with functional annotation in the PHI database; **c** CAZymes

Based on normalized expression values calculated by RNA-seq analysis, hierarchical clustering of expression patterns by heatmap analysis revealed that *in planta* genome-wide transcriptional profiles were more similar to each other than to the profiles at the aeciospore and urediniospore stages; likewise, the latter two showed greater similar to each other. Similar patterns were observed among three categories of genes encoding secreted proteins, candidate effectors, and CAZymes (Fig. 5). These results demonstrate that the Cri transcriptome is extensively reprogrammed during pine stem infection.

Due to their functional relevance to virulence and pathogenicity, secreted proteins, candidate effectors, and CAZymes were examined in detail for regulation of their transcript expression by heatmap analysis. Of 734 secreted protein genes, 51.5 % of them were identified as DEGs and the heatmap showed six transcript expression patterns: I, III, and VI with up-regulation at rust spore developmental stages, and II, IV, and V with *in-planta* up regulation (Fig. 5a). Genes encoding secreted proteins in pattern V exhibited up-regulation in both infected pine needles and cankered stem. DEGs accounted for 29.7 % of 881 annotated Cri candidate effectors that encompassed all six transcript expression patterns, among them patterns II, III-b, IV, and V show transcripts with *in-planta* up-regulation (Fig. 5b). 43.5 % of Cri CAZymes were identified as DEGs and they were clustered into four expression patterns by heatmap analysis (Fig. 5c). Although many CAZymes were highly expressed in urediniospores, CAZyme genes in subclusters I-a, II-b, II-d, and II-f were significantly up-regulated only in the cankered stems (Fig. 5c).

The proteins belonging to fungal gene families with multiple members were generally distributed in different clusters or subclusters on the expression heatmaps. Some members were expressed at similar levels in the fungal cells from both infected pine stems and *Ribes* leaves while other members were expressed in contrasting patterns between these two infected hosts (Additional file 1: Table S9). As exceptions to this general observation, members of seven CAZy families (CE8, GH2, GH16, GT2, GT26, GT31, and GT43) and proteins with CFEM, ferritin_2, zf-C2H2, and RVT-1 domains were highly expressed in infected *Ribes* leaves. In contrast, gene families for DnaJ-X/HSP90, FKBP, peptidase-S10, superoxide dismutases (Fe-SOD), β-1,3-glucanosyltransglycosylases (GH72), ATP synthases (ATP-synt_ab), the cysteine-rich secretory proteins, antigen 5, and pathogenesis-related 1 proteins (CAP), citrate synthases (Citrate_synt), pectate lyases (Pec_lyase_C), and polysaccharide lyases (PL1/PL3) were highly expressed in infected pine stems (Additional file 1: Table S9).

To validate whether the RNA-seq analysis reflected their gene expression, 21 genes were selected as representatives of secreted proteins, CAZymes, and PHI proteins and they were used to perform qRT-PCR analysis (Additional file 1: Table S10). Relative expression values were highly variable among biological repeats, but this qRT-PCR analysis confirmed the general expression patterns as revealed by RNA-seq analysis (Additional file 1: Table S11). Additional file 4: Figure S3 shows that the fold changes of transcript expression levels measured by qRT-PCR and RNA-seq analyses were highly correlated (Pearson correlation R^2^ > 0.94) with statistical significance (*p* < 0.00001).

### DEGs up-regulated in infected pine stem

**Fig. 6:**
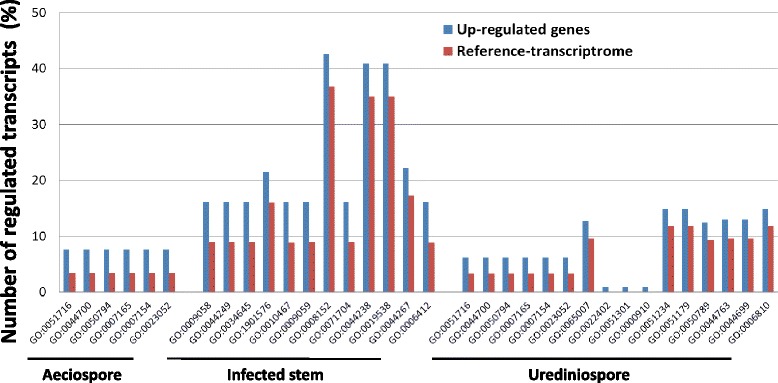
Enrichment analysis of gene ontology (GO) terms for up-regulated genes at three *Cronartium ribicola* life cycle stages: aeciospore, urediniospore and *inplanta* mycelium growth in infected *Pinus monticola* stem tissues. Fisher’s test was performed using Cri reference transcriptome as reference (p <0.05 with FDR correction). GO terms involved in biological process include: GO:0051716, cellular response to stimulus; GO:0044700, single organism signaling; GO:0050794, regulation of cellular process; GO:0007165, signal transduction; GO:0007154, cell communication; GO:0023052, signaling; GO:0009058, biosynthetic process; GO:0044249, cellular biosynthetic process; GO:0034645, cellular macromolecule biosynthetic process; GO:1901576, organic substance biosynthetic process; GO:0010467, gene expression; GO:0009059, macromolecule biosynthetic process; GO:0008152, metabolic process; GO:0071704, organic substance metabolic process; GO:0044238, primary metabolic process; GO:0019538, protein metabolic process; GO:0044267, cellular protein metabolic process; GO:0006412, translation; GO:0065007, biological regulation; GO:0022402, cell cycle process; GO:0051301, cell division; GO:0000910, cytokinesis; GO:0051234, establishment of localization; GO:0051179, localization; GO:0050789, regulation of biological process; GO:0044763, single-organism cellular process; GO:0044699, single-organism process; GO:0006810, transport

To understand the potential roles of DEGs in biological process, Fisher’s test (FDR *P* < 0.05) for enrichment of GO terms was performed using the Cri reference transcriptome as a reference data set. Compared to either aeciospores or urediniospores, the infected stem showed up-regulated DEGs with enriched GO terms of biosynthetic process (five GO terms), metabolic process (five GO terms), gene expression and translation (two GO terms) (Fig. 6). The DEGs contributing to enriched biosynthetic processes include a large number of genes encoding various ribosomal proteins, histones, transcriptional factors, and enzymes for biosynthesis of macromolecules (DNA, RNA, and proteins), amino acids, fatty acids, lipids, steroids, sugar, ATP, and others (Additional file 1: Table S12). Protein synthesis from transcription to posttranslational modification seems to be a major process in the infected pine stem tissues. Among multiple families of up-regulated transcription factors, fork-head box (FOX) proteins are a family of transcription factors that play important roles in regulating the expression of genes involved in cell growth, proliferation, differentiation, and longevity. Active protein synthesis was further evidenced by the strong induction of many ribosomal proteins and other components in translation. The up-regulated metabolic genes were largely involved in DNA and carbohydrate metabolism. A number of transcripts associated with transposons and retrotransposons were also detected to be over-presented in infected stem tissues (Additional file 1: Table S12).

### DEGs up-regulated at life cycle stages of aeciospore and urediniospore development

Compared with infected stems, 1,191 transcripts were up-regulated at the aeciospore stage. GO term enrichment analysis by Fisher’s test showed aeciospore-upregulated DEGs participated in signal transduction/response to stimulus (six GO terms) (Fig. 6). A few main groups of DEGs encode for small GTPases, MAPK, and histidine kinases (Additional file 1: Table S12).

Similarly, there were 2,082 transcripts up-regulated at the urediniospore stage compared to infected stems. Fisher’s test identified 16 GO terms enriched in these urediniospore-upregulated DEGs (Fig. 6). Apart from genes in signal transduction pathways, other urediniospore-upregulated DEGs were involved in cell division/cytokinesis, regulation of biological processes, and transport/localization. Transport families were one of the major components of the urediniospore transcriptome profile, 31 of which were upregulated during urediniospore development (Additional file 1: Table S12).

Compared with aeciospores, 302 DEGs were developmentally up-regulated at the urediniospore stage. GO analysis revealed that these DEGs have putative functions in organic substance metabolic process, nitrogen compound metabolic process, biosynthetic process, regulation of biological process, establishment of localization, cellular response to stimulus, cellular component biogenesis, single organism signalling, and single-organism developmental process (Additional file 1: Table S13).

### Identification of Cri genes expressed at the early stage of needle infection

RNA-seq data from basidiospore-infected WWP needles at 4 dpi was used to identify Cri genes expressed in compatible (*cr2/cr2* vs. *avcr2*) and incompatible (*Cr2/-* vs. *avcr2*) WP-BR interactions. A total of 1,722 and 818 transcripts were detected in susceptible (*cr2/cr2*) and resistant (*Cr2/-*) needles at 4 dpi respectively (Fig. 4), including transcripts encoding 145 unique secreted proteins. A Z-test revealed 25 transcripts with expression levels significantly higher in resistant needles than in susceptible needles. Although they were not well annotated, six of them encoded proteins with distinct domains (including one with signal peptide) for potential functions as identified by InterProScan in the BLAST2GO program. In contrast, 234 transcripts were detected with expression levels significantly higher in compatible (*cr2/cr2* vs. *avcr2*) interactions than in incompatible (*Cr2/-* vs *avcr2*) interactions, 83 of which shared homology with sequences in the NCBI nr database and 44 of which were assigned GO terms (Additional file 1: Table S14). These annotated DEGs encoded histones, cyclins, secreted proteins with unknown function (including one Hesp-379-like protein), CAZymes (GH5/16/17/23/26; GT32; CE4; and CBM13), proteases, expansins, and enzymes involved in oxidation-reduction process. A large number of these DEGs are known to be involved in molecular modification of cell walls.

## Discussion

### *In planta* RNA-seq analysis of the WPBR pathosystem

In this study we generated genome-wide transcriptome and secretome profiles of *C. ribicola* at three stages of its life cycle. To our knowledge, this is the first report of the pathogen transcriptome and dual transcriptomes of pine tissues infected by *C. ribicola* using RNA-seq. In the present study we used an approach of consensus assembly among biological repeats for each sample to remove potentially contaminated raw data for generation of a reference transcriptome. High mapping ratio of RNA-seq reads (>85 %) to the reference transcriptome indicated that this data processing approach using rust fungal spore samples was satisfactory for *de novo* assembly of a biotrophic pathogen transcriptome without a reference genome sequence. For biotrophic fungi such as Cri, using spores for genomics research is much more straightforward than using *in-vitro* cultures, which tend to be recalcitrant and/or have extremely slow growth rates. BLAST and GO analyses showed that the *de novo* assembled transcriptome is almost complete, being similar in size to the recently reported transcriptomes of other rust fungi [11, 29]. Profiling of the Cri transcriptomes revealed that the majority of expressed genes (>76 % of the reference transcriptome) were detectable at the development stage of either aeciospore or urediniospore. Over 60 % of the reference transcriptome was expressed at all three life cycle stages analysed in this study.

Reassembly of unmapped RNA-seq reads from fungal spore cDNA libraries using the CLC program showed that 31.7 % of them remained as singletons, and others were *de-novo* re-assembled into ~51,000 short contigs (N50 = 540 bp and average length = 492 bp). The largest proportion (47.3 % of the total) of the reassembly might have originated from aphids, some may have fungal origin (32.0 % of the total), and other short contigs likely represent a set of contaminant sequences from host plants (8.1 % of the total) and bacteria (6.7 % of the total) as revealed by BLASTx analysis at E cut off value of e^-5^ (data not shown). Aphid-like sequences appeared to be associated with some rust-infected Ribes leaves where urediniospore samples were collected for RNA-seq analysis. Reassembly analysis of unmapped reads suggests that extraction of consensus sequences as a reference transcriptome may be an effective strategy for removal of contamination in RNA-seq analysis of field or green-house biosamples.

Without known reference sequences for host and pathogen genomes, *in planta* transcriptome analysis is a challenging task [30], especially for the WPBR pathosystem with alternate hosts and a long infection process that can take up to a few years. RNA-seq based dual transcriptome profiling has been recently reported on rice blast [31] and target leaf spot of sorghum [32]. Although infected pine stems exhibited a wide variation of disease symptoms, our RNA-seq analysis demonstrated a good reproducibility of the gene expression patterns among sample replicates harvested from multiple infected seedlings. Analysis of dual transcriptomes in infected stems revealed a similar coverage of the rust fungal transcriptome as those at spore developmental stages, suggesting that *C. ribicola* is physiologically active inside the infected host tissues. These results demonstrate that dual transcriptome analysis is efficient in the WPBR pathosystem, and more secreted proteins and candidate effectors were detected in the infected stem than in spore samples. This research strategy also allowed comparison of transcriptome profiles and further identification of DEGs regulated *in planta* during mycelial growth, which may be applicable to other biotrophic conifer pathosystems.

### *C. ribicola* candidate effectors

Numerous proteins secreted by pathogens are known to function as effectors in modulating host defence and metabolism [8]. An effector displays a property of either virulence or avirulence, depending on whether the host plant processes a corresponding immunoreceptor that recognizes the effector. Effectors enable pathogens to successfully colonize plant tissues and facilitate disease, but they function as Avr factors when the host acquires discrete recognition capabilities that trigger immunity during an incompatible plant-microbe interaction. A repertoire of effectors is believed to collectively suppress plant basal innate immunity through escaping detection of pathogen-associated molecular patterns (PAMPs) by specific host receptors, and also to constitute a favourable intercellular habitat for mycelium growth and disease development in susceptible host tissues during compatible plant-microbe interaction [33].

Corresponding to major resistance (R) genes (*Cr1-Cr4*) in different white pine species [1], four Cri Avr effectors, avcr1-avcr4, are proposed to be recognised by immunoreceptors encoded by the white pine R genes (*Cr1-Cr4*). When infected by an *avcr* race, the needle tissues at local infection sites exhibit HR featured by rapid programmed cell death in the white pine trees carrying an R gene. In contrast, virulent races (*vcr1* and *vcr2*) overcome sugar pine *Cr1* and western white pine *Cr2*, respectively [4]. Effectors vcr1 and vcr2 are hypothesized to allow Cri races to avoid detection by the white pine’s R genes (*Cr1* or *Cr2*), resulting in successful infection of white pine seedlings with MGR.

Most fungal effectors are SSPs shorter than 300 amino acids in length and rich in cysteine [33, 34]. A Cri SSP was found to be accumulated during white pine infection [35]. However, so far no Cri effector has been identified at the molecular level. Recently, a few rust fungal effectors have been identified, including four Mli Avr proteins [36], Pgt AvrSr22 [37], and *Uromyces fabae* transferred protein 1 (RTP1) [38, 39]. Compared with these identified rust fungal effectors, only three Cri secreted proteins (SB-Spr-contig228, SB-Spr-contig262, and Spr121122) were identified as RTP1-homologs. RTP1 homologues were reported in at least 13 other rust fungi, suggesting that this protein family may play a universal role in biotrophic rust fungi [39]. However, whether Cri RTP1-homologs have a similar function as protease inhibitor still awaits a further verification.

In this study 734 unique secreted proteins were predicted in the Cri transcriptome; which is considerably fewer than those reported in Mlp, Pgt, and *C. quercuum* f. sp. *fusiforme* genomes [11, 40]. Because ~46 % of the putative protein sequences in the Cri reference proteome were incomplete, we were unable to identify a complete set of Cri secreted proteins in the present study likely due to partial sequences and missed signal peptides. To predict candidate effectors, we used a similar strategy as reported by Nemri et al. [13]. Using this approach with a further search using the PHI dataset, we catalogued a total of 2,770 proteins as candidate effectors in the first draft of the Cri proteome; much less than that reported for the other four rust fungi [13]. As the PHI dataset expands, it is likely that bioinformatics analysis will allow additional candidate effectors to be mined from the genomes or transcriptomes of pathogens. Among 2,770 Cri candidate effectors, 31.8 % of them were characterized using Pfam domain annotation and assignment of potential properties due to similarities to known effectors and pathogenicity-related proteins (Additional file 1: Table S5).

Among the first draft of Cri secretome with 734 unique secreted proteins, only 267 (36.4 % of the total) possessed distinct Pfam-A domains with some functional annotation. Similar to secretomes of other biotrophic fungi [41, 42] the Cri secretome consisted of two main groups: degrading hydrolases (such as secreted CAZymes, secreted proteases, and secreted lipases) (Additional file 1: Table S4) and putative effectors with unknown function. Using a set of candidate effectors available from other rust fungi [13], we compared Cri with them and identified 301 *Cronartium*-specific secreted proteins (41 % of the total). Other secreted proteins are conserved among rusts, a large part of which have well-characterized domains, such as proteases, protease inhibitors, and CAZymes. These conserved secreted proteins may provide a valuable resource for further understanding evolution of rust pathogenicity.

The lineage-specific secreted proteins, especially those identified as SSPs, may contain candidate effectors that are likely to be enriched as determinants of host specificity [43]. Although most fungal SSPs have no functional homology in databases available to date, their sequence features (i.e. presence of signal peptide, small size, cysteine-rich, additional conserved motifs, and expression patterns) have been widely used for computational prediction of candidate effectors in fungal genomes or transcriptomes [10–13, 44–46]. Of all the Cri secreted proteins we found, 155 were SSPs. Even though about 82 % of these had no GO term hits in a BLAST2GO search, most of them were expressed *in planta*, suggesting that they may be specific and essential for the pathogenicity and virulence of *C. ribicola*. Those Cri lineage-specific SSPs provide a repertoire of candidate effectors for molecular identification of vcr or avcr effectors in a future study.

### Regulatory programming of gene expression during host infection

Genome-wide transcriptional profiling has provided a powerful approach to reveal *in planta* regulation of pathogen virulence factors [45, 47–50]. In order to provide insights into how the fungus programs its biological processes to cause WPBR, we characterized the Cri transcriptome within infected pine stems, noting dynamic changes of expression profiles during infection and disease development relative to expression in the spores. We found that rust fungal genes up-regulated during infection of pine stems were involved in biosynthesis all the way from DNA replication/ metabolisms, nucleosome assembly, RNA transcription, protein translation, to down-stream biosynthesis of various metabolites (steroids, lipids, fatty acids, and others).

We observed several transposons, retrotransposons, helicases, and RNA-directed DNA polymerases were up-regulated in infected stems (Additional file 1: Table S12). Gene regulation is even more important than genetic polymorphism for understanding the source of phenotypical virulence diversity in some pathogens. *Phytophthora ramorum* transposable elements showed an isolate-dependent expression pattern, and elevated expression of transposable elements was associated with a ‘non-wild’ phenotype [51]. Transposable elements and the DNA repair system have a dramatic impact on genomic diversity [52]. Diversity at genomic regions enriched with transposons is one of mechanisms for rapid evolution of novel effectors in pathogenic microbes [53]. Given that rust mycelium may grow several years inside pine bark tissues, enhanced activities of transposons and retrotransposons may allow these genetic elements to play a pathogenicity-related role in canker disease development.

A series of transcriptional factors showed increased expression in both infected pine stems and infected *Ribes* leaves, but different gene families or different members of the same families were involved in the regulatory networks specific to each host (Additional file 1: Table S12). This suggests that differential expression of transcriptional factors with co-ordinated functions may be a regulatory mechanism for the rust fungus to fine tune its growth and development on different hosts. Furthermore, regulatory programming in infected *Ribes* was integrated with many other signalling components, probably up-stream of transcriptional factors, including histone modification, GTase-mediated signalling, MAPK cascade, etc. Like genomes of other rust fungi [54], we identified a large variety of gene families coding for signal transduction pathways in *C. ribicola*. Among these signalling gene families, the superfamily of protein kinases was most abundant in the repertoire of Cri candidate effectors. Protein kinases function in many cellular processes, including metabolism, transcription, cell cycle progression, cytoskeletal rearrangement and cell movement, apoptosis, and differentiation. A non-pathogenic *U. maydis* mutant was restored its pathogenicity by complementation with *P. triticina* PtMAPK1 [55]. This highlights the importance of energy dependent signaling cascades during *Ribes* infection, supporting the assumption that these gene families are involved in signal perception mechanisms during rust fungal urediniospore development [11].

### *C. ribicola* gene expression at early stages of infection in pine needle

Rust fungal genes highly expressed at the early needle infection stage may be of pathogenic importance for establishment of infection colonies inside host tissues. Using RNA-seq data generated previously [56], we detected difference of rust fungal gene expression between compatible (*cr2/cr2* vs. *avcr2*) and incompatible (*Cr2/-* vs. *avcr2*) interactions at 4 dpi. Although mapped reads (11 million vs. 6 million) were almost double in resistant needles compared to susceptible needles, the number of transcripts detected in susceptible needles (1,772 vs. 818) was more than double that in resistant needles.

Because only ~6 % of total RNA-seq reads were derived from expressed rust fungal genes in the infected pine needles at an early infection stage (4 dpi), it could not be compared with other stages of the rust life cycle (Table 1). Using infected pine needles, we compared rust fungal gene expression between compatible and incompatible interactions. DEGs significantly upregulated in susceptible needles included a high number of transcripts encoding cell wall-degrading/modifying enzymes such as CAZymes (GH5, 16, 17, 23, and 26; GT32; CE4; CBM13), proteases, expansin, copper radical oxidases, and a lot of other secreted proteins with unknown function. Most enzymes that act on components of the plant cell wall belong to the GH superfamily. GHs are increasingly being documented as virulence factors in pathogens, including enzymes of nine GH families (GH2, 13, 18, 20, 30, 33, 73, 84, and 101) [57]. At least 44 GH families are reported in fungi [58]. During the early stages of WP-BR interactions, members of five GH families and one CMB family were significantly induced, including exo-β-1,3-glucanase (GH5), endo-1,3-β-glucanase (GH16), endo-1,3-β-glucosidase (GH17), peptidoglycan lyase (GH23), β-1,3-xylanase (GH26), and CMB13 with putative binding activities to GHs, GTs, xylan, and lectins. These enzymes may modify components of pine cell walls, making fungal penetration easier at the initial stages of needle infection. This result showed that degradation of plant cell macromolecules (such as cellulose, hemicellulose and polysaccharides) is a key process for *C. ribicola* to invade pine needle tissues. Various GH enzymes also have the potential to modify the cell wall of the fungus itself, reducing the effectiveness of the plant enzymes or preventing the elicitation of plant defense responses. For example, CE4 genes encode carbohydrate esterases that deacetylate polymeric carbohydrate substrates such as chitin, acetyl xylan and peptidoglycan. Deacetylation of peptidoglycan is a mechanism used by pathogens to evade innate host defenses [59].

Serine proteases are associated with virulence and nutrient cycling in many pathogens. *Magnaporthe grisea* mutant of a serine protease gene (*spm1*) displays phenotypes of decreased sporulation and appressorial development as well as a greatly attenuated ability to cause disease [60]. As one of the most abundant domains in the Cri secretome, the domain DPBB-1 is found in fungal expansins. Concomitantly, two Cri expansin genes were highly induced in infected susceptible needles, consistent with a previous report that found expression of *P. monticola* expansins highly down-regulated in resistant needles post rust infection [56]. Expansins are well-known in loosening cell walls by inducing the slippage of cellulose micro fibrils. Loosening the cell wall is necessary for plant growth, but it also makes the plant vulnerable to pathogen attack. Expansins induced by pathogen-secreted indole-3-acetic acid (IAA) increase rice disease symptoms [61]. A *Trichoderma* expansin is involved in colonization of plant roots and apparently elicits plant defense responses [62]. Of 540 candidate effectors detected at early needle infection stage, many of them were induced as predicted secreted proteins but with unknown function. In general, apoplastic effectors are SSPs with primarily inhibitory effects on host proteases, hydrolases, glucanases, and other lytic enzymes, protecting the pathogen cell wall or neutralizing antimicrobial molecules released during the host defense response [63]. Cell wall modification by CAZymes, proteases, expansins, and other proteins seems important for successful fungal colonization in the WPBR pathosystem.

Other annotated DEGs up-regulated during the early stages of needle infection include cyclin, glutathione-S-transferases (GST), cytochrome P450-like TATA box binding protein (TBP), and copper radical oxidase (CRO). Cyclins control the progression of cells through the cell cycle by activating cyclin-dependent kinase enzymes, and GSTs represent an extended family involved in detoxification processes. TBP plays important endogenous and exogenous roles in oxidative metabolism [64], and CRO contributes to extracellular peroxide production, which is linked to hyphal growth and pathogenicity [65]. Such DEGs may participate in the Cri response to reactive oxygen species released by white pine needle cells as a host defense mechanism. Overall, enhanced expression implies that these DEGs play a role in compatible WP-BR interactions, most likely by manipulating host defense (such as suppression of oxidative burst) and assisting migration through the host tissues.

## Conclusion

*De novo* sequencing of the Cri transcriptome has been employed to identify Cri proteins involved in compatible WP-BR infections. Global expression profiling of the Cri transcriptome, secretome, and effectorome provides novel insights into the molecular pathogenicity of this important forest biotrophic fungus. RNA-seq analysis revealed DEGs at an early infection stage in the needles and at a later infection stage in pine stem cankers. A significant portion of the identified DEGs were catalogued as candidate effectors, especially those predicted to be secreted proteins. These candidate effectors potentially function in modification of the host cell wall to suppress plant defenses, allowing the rust pathogen to colonize susceptible needle tissues. Up-regulation of other rust fungal proteins in the infected stem indicates that the rust fungus has well adapted to the mycelium growth habitat by actively producing enzymes for biosyntheses of various cellular components. These findings support the existing biotrophic pathogenesis model for the WPBR pathosystem, and present a suite of putative effectors potentially functioning at various stages of the rust life cycle, as well as information regarding the differential use of unique members of complex gene families.

Because a high number of genes are *Cronartium*-specific and expressed as hypothetical proteins during the disease process, further research is needed to fully understand their involvement in pathogenicity. A future study may focus on identifying variants of Cri candidate effectors by global comparison of transcriptomes between *avcr* and *vcr* races. As more genetic lineages among populations of this rust fungus are identified, more races will be sequenced to explore association of non-synonymous or functional SNPs of the candidate effectors with virulence levels. Potential pathogenic importance of candidate effectors may be dissected by evolution and association genetics analyses, which will narrow down the number of candidates for functional verification to determine which ones play a key role in resistant or susceptible responses of five-needle pines. Characterization of Cri effectors will allow a more comprehensive understanding of how rust fungal effectors are recognized by R proteins in white pines. With candidate R genes identified in WWP [23], a final characterization of molecular determinants in disease development will provide effective management tools for operational application in breeding programs of five-needle pines as well as for rating of rust hazard in forest ecosystems.

## Methods

### Fungal and plant materials

*C. ribicola* aeciospores were collected from infected WWP stems before blisters were broken from Coombs, Vancouver Island, British Columbia, Canada in May 2013, and they were used to inoculate black currant (*Ribes nigrum*, cultivar Ben Nevis). When urediniospores began to develop, rust samples (including urediniospores as well as mycelium) were collected from *Ribes* leaf surfaces. Infected stems showing disease symptoms of discoloured bark tissues were collected from 20-month-old susceptible (*cr2/cr2*) WWP seedlings ~14 months post *C. ribicola* infection as described previously [23]. All rust isolates were avirulent (*avcr2*) and each sample was harvested with three biological repeats using liquid nitrogen and stored at −80 °C before RNA extraction.

### RNA-seq analysis and *de novo* transcriptome assembly

Total RNAs were extracted from rust samples and infected host tissues following a protocol described previously [56]. Messenger RNA (mRNA) was separated using an RNA-seq sample preparation kit (Illumina). cDNA libraries were constructed with specific 6-bp nucleotide bar-coding tags. Tagged cDNA libraries were pooled in equal ratios and used for 100-bp PE sequencing on the Illumina HiSeq2000 instrument (Illumina, San Diego, CA, USA) at the National Research Council of Canada (Saskatoon, Canada). The raw Illumina RNA-seq 100-bp PE sequences of *C. ribicola* spores were deposited in the NCBI SRA under accession number SRR1583557-1583540, SRR1583545, and SRR1583552.

Raw reads were first trimmed using Trimmomatic with default settings at ILLUMINACLIP:TruSeq3-PE.fa:2:30:10 LEADING:3 TRAILING:3 SLIDINGWINDOW:4:15 MINLEN:36 [66]. As infected white pine tissues contained both host and rust fungal transcriptomes and rust samples (aeciospores and urediniospores) contained only fungal transcriptomes, their RNA-seq reads were assembled separately. We used the trimmed reads from six cDNA libraries of aeciospores and urediniospores to generate a preliminary assembly by *de novo* assembly using Trinity (version: trinityrnaseq_r2013-02-25) with default *k*-mer length of 25 [67]. Following a preliminary assembly of RNA-seq reads, contigs were designated as consensus transcriptome sequences if they shared at least one unique mapping read in each of three biological repeats for each sample, and a foreign contamination screen was used to filter bacterial-, animal-, or plant-like sequences from the dataset using the NCBI BLAST pipeline at a cut-off of 100 nt and 98 % identity. The resulting transcript dataset was considered as a Cri reference transcriptome for further analysis, and it has been deposited at DDBJ/EMBL/GenBank under accession GBSG01000000 and the BioProject ID PRJNA261951.

To verify *de novo* assembly quality, putative open reading frames (ORFs) were identified within transcripts by TransDecoder, which is integrated into Trinity, at minimum protein length of 50. Reciprocal BLASTp analysis was performed among the proteomes of *C. ribicola* and other rust fungal species: Mlp with16,399 proteins [11], Mli with 26,443 proteins [13], Pgt with 20,534 proteins [11], and Pst with 18,023 proteins [10, 12]. Gene annotation was performed using BLAST2GO [68]. Gene names and gene ontology (GO) terms were assigned to the Cri genes based on their homologies to the available databases (NCBI-nr, PIR, KEGG, and GO).

### Prediction of secreted proteins

Complete ORFs were scanned for signal peptides using signalP v4.1 at a D-cut-off value of 0.36 [69]. The resulting peptides then were scanned for transmembrane helices and mitochondria-targeted sequence using the TMHMM program v2.0 [70] and TargetP v1.1 [71], respectively. To find candidate effectors from partial ORFs, the Cri proteome was used as a query for BLASTp against the Cri secreted proteins predicted from complete ORFs, a dataset of candidate effectors of Mpl, Mli, Pst and Pgt [13], and the pathogen-host interaction database (PHI-base v3.2) containing experimentally verified pathogenicity, virulence and effectors from fungal, oomycete, and bacterial pathogens [28]. Putative CAZymes were identified by BLAST against CAZyDB (3/22/2013) using the dbCAN v3.0 HMM-based CAZy annotation server [25]. A Pfam search was performed using on-line service from The European Bioinformatics Institute (EMBL-EBI) at gathering threshold with a cut-off of e^-3^ and dom of e^-3^. Assignment of gene families was performed by BLASTp against OrthoMCL proteins at a cut-off of e^-5^, and 50 % match using OrthoMCL-5 [72]. Similarly, proteins involved in membrane transport were investigated by a BLASTp search against predicted polypeptides in the Transporter Classification Database [27].

### Global gene expression analysis

Transcriptome profiles were compared at four stages of the Cri life cycle: aeciospores, urediniospores, infected needles (4 dpi) of six-months-old *P. monticola* seedlings, and cankered stems (~14 months post basidiospore infection) of 20-month-old *P. monticola* seedlings. RNA-seq reads from infected pine stems were used to identify *in planta* differentially expressed genes (DEGs) by comparison with RNA-seq reads from aeciospores and urediniospores. Trimmed reads from each sample were mapped to the Cri reference transcriptome and only sequence pairs (fragments) were counted in read mapping with a minimum length fraction of 0.9 and a minimum similarity fraction of 0.9 and expression values were calculated as FPKM (Fragments Per Kilobase of exon per Million fragments mapped) using CLC Genomics Workbench 5.5 (CLC bio, QIAgen, Aarhus, Denmark). Gene expression values (FPKM) were normalized with parameters: normalization method = scaling, scaling method = mean, trimming = 5 %, normalized data = original expression values. Statistical analysis was performed with parameters: statistical test = t-test, variances = homogeneous, comparisons = all pairs, use pairing = no, data to analyze = normalized expression values, add FDR correction p-values = yes. Transcripts with normalized fold change > ±2 at *p* <0.05 after adjustment with false discovery rate (FDR) were extracted as up- or down-regulated DEGs.

RNA-seq data of Cri-infected *P. monticola* primary needles at 4 dpi was generated in a previous study [56] and here used to investigate rust fungal gene expression in compatible (*cr2/cr2* vs. *avcr2*) and incompatible (*Cr2/-* vs. *avcr2*) interactions. Statistical analysis was same as above except that a Z-test was used as described previously [56]. Hierarchical clustering and heatmap production for the expression profiles were performed using the gplots heatmap.2 package in R 3.1.2 [73] with the data stripped of records where the normalized expression values were all zero (0). Cluster agglomeration was performed using Manhattan distance and intracluster distance set to complete.

### Quantitative reverse-transcriptase PCR (qRT-PCR) analysis

To confirm gene expression levels measured by FPKM fold change in RNA-seq analysis, 21 DEGs from RNA-seq analysis were selected due to their putative roles in virulence or pathogenicity for qRT-PCR analysis. Primers were designed using Primer3 software, and *α-tubulin* was used as an internal control. Three biological replicates and two technical replicates were run on an Applied Biosystems 7500 Fast Real-time PCR System (Life Technologies). Healthy *P. monticola* tissues, no reverse-transcriptase and water samples were run as controls for each primer pair.

Expression Suite Software v1.0.3 (Life Technologies) was used to analyze relative expression levels of each gene with aeciospore samples as the reference. Relative quantification (RQ) was calculated as 2^-ΔΔCt^. Student t tests were used to analyze the significance of transcript differences between two samples. Correlation and regression analyses were performed to compare fold changes of transcripts measured by qRT-PCR and RNA-seq analyses with ANOVA tests for statistical significance.

### Availability of supporting data

All sequencing data generated in this study is available from the SRA-Archive (http://www.ncbi.nlm.nih.gov/sra) under the study accession SRP031625. Illumina raw sequences were deposited in the NCBI GenBank under accession number SRR1583557-1583540, SRR1583545, and SRR1583552. A *Cronartium ribicola* reference transcriptome was deposited at DDBJ/EMBL/GenBank under accession GBSG01000000.

All other supporting data are included as additional files.

## Additional files

Additional file 1: Table S1. Statistical characteristics of *de-novo* assembly of *Cronartium ribicola* transcriptomes. **Table S2.** Open reading frames (ORFs) predicted by TransDecoder for the Cri proteome. **Table S3.** BLAST analysis of *Cronartium ribicola* transcript and protein sequences against other databases. **Table S4.** Annotation and classification of *Cronartium ribicola* carbohydrate-active enzymes. **Table S5.** Classification of *Cronartium ribicola* transporter proteins. **Table S6.** HMM domain identification of *Cronartium ribicola* secreted proteins. **Table S7.** *Cronartium ribicola* candidate effectors with annotation of Pfam domains and putative functions in pathogenicity. **Table S8.** Expression data (FPKM) of the *Cronartium ribicola* reference transcriptome based on RNA-seq analysis. **Table S9.** Annotated *Cronartium ribicola* genes with opposite expression patterns between cankered stems and infected *Ribes* leaves at urediniospore stage. **Table S10.** qRT-PCR primers for analysis of *Cronartium ribicola* genes. **Table S11.** Evaluation of transcript expression level by qRT-PCR. Gene expression level was calculated as relative quantification (RQ) using ABI ExpressionSuite software. **Table S12.** Gene ontology (GO) enrichment analysis of differentially expressed genes (DEGs). **Table S13.** Gene ontology (GO) analysis of differentially expressed genes (DEGs) up-regulated in urediniospores relative to aeciospores. **Table S14.** BLAST and GO analyses of differentially expressed genes (DEGs) up-related in early compatible WPBR interactions relative to incompatible WPBR interactions. (PPTX 2770 kb) Additional file 2: Figure S1. Top-hit species distribution for the *Cronartium ribicola* transcripts measured by BLASTx analysis using the BLAST2GO program. (PPTX 143 kb) Additional file 3: Figure S2. Functional classification of the *Cronartium ribicola* reference transcriptome assembled *de novo* from RNA-seq data based on gene ontology (GO). Subcategories of biological process, molecular function, and cellular component are indicated as: GO:0008152, metabolic process; GO:0009987, cellular process; GO:0044699, single-organism process; GO:0050896, response to stimulus; GO:0051179, localization; GO:0065007, biological regulation; GO:0071840, cellular component organization or biogenesis; GO:0032502, developmental process; GO:0023052, signaling; GO:0032501, multicellular organismal process; GO:0000003, reproduction; GO:0040007, growth; GO:0051704, multi-organism process; GO:0040011, locomotion; GO:0002376, immune system process; GO:0022610, biological adhesion; GO:0048511, rhythmic process; GO:0001906, cell killing; GO:0005488, binding; GO:0003824, catalytic activity; GO:0005198, structural molecule activity; GO:0005215, transporter activity; GO:0009055, electron carrier activity; GO:0001071, nucleic acid binding transcription factor activity; GO:0016209, antioxidant activity; GO:0030234, enzyme regulator activity; GO:0060089, molecular transducer activity; GO:0000988, protein binding transcription factor activity; GO:0004872, receptor activity; GO:0045735, nutrient reservoir activity; GO:0016530, metallochaperone activity; GO:0005623, cell; GO:0043226, organelle; GO:0032991, macromolecular complex; GO:0016020, membrane; GO:0031974, membrane-enclosed lumen; GO:0005576, extracellular region; GO:0009295, nucleoid; GO:0030054, cell junction; GO:0045202, synapse; GO:0031012, extracellular matrix; GO:0055044, symplast. (PPTX 104 kb) Additional file 4: Figure S3. Scatter plot of relative levels of transcript expression as measured by RNA-seq and qRT-PCR. Relative levels of transcript expression are presented as log2 (fold-change). Both RNA-seq and qRT-PCR data were analyzed based on three biological replicates. Pearson coefficient correlation analysis between data from RNA-seq and qRT-PCR shows R^2^ = 0.94 (p-value < 0.00001). (XLSX 6464 kb)
