# Supplementary material for: Transcriptome analysis of the white pine blister rust pathogen Cronartium ribicola: de novo assembly, expression profiling, and identification of candidate effectors
Source: BMC Genomics. 2015 Sep 4;16(1):678. doi: 10.1186/s12864-015-1861-1 (PMC4559923; doi:10.1186/s12864-015-1861-1)
Supplement: Additional file 1: Table S1. — Statistical characteristics of de-novo assembly of Cronartium ribicola transcriptomes. Table S2. Open reading frames (ORFs) predicted by TransDecoder for the Cri proteome. Table S3. BLAST analysis of Cronartium ribicola transcript and protein sequences against other databases. Table S4. Annotation and classification of Cronartium ribicola carbohydrate-active enzymes. Table S5. Classification of Cronartium ribicola transporter proteins. Table S6. HMM domain identification of Cronartium ribicola secreted proteins. Table S7. Cronartium ribicola candidate effectors with annotation of Pfam domains and putative functions in pathogenicity. Table S8. Expression data (FPKM) of the Cronartium ribicola reference transcriptome based on RNA-seq analysis. Table S9. Annotated Cronartium ribicola genes with opposite expression patterns between cankered stems and infected Ribes leaves at urediniospore stage. Table S10. qRT-PCR primers for analysis of Cronartium ribicola genes. Table S11. Evaluation of transcript expression level by qRT-PCR. Gene expression level was calculated as relative quantification (RQ) using ABI ExpressionSuite software. Table S12. Gene ontology (GO) enrichment analysis of differentially expressed genes (DEGs). Table S13. Gene ontology (GO) analysis of differentially expressed genes (DEGs) up-regulated in urediniospores relative to aeciospores. Table S14. BLAST and GO analyses of differentially expressed genes (DEGs) up-related in early compatible WPBR interactions relative to incompatible WPBR interactions. (PPTX 2770 kb) [file 12864_2015_1861_MOESM1_ESM.pptx]

## Slide 1
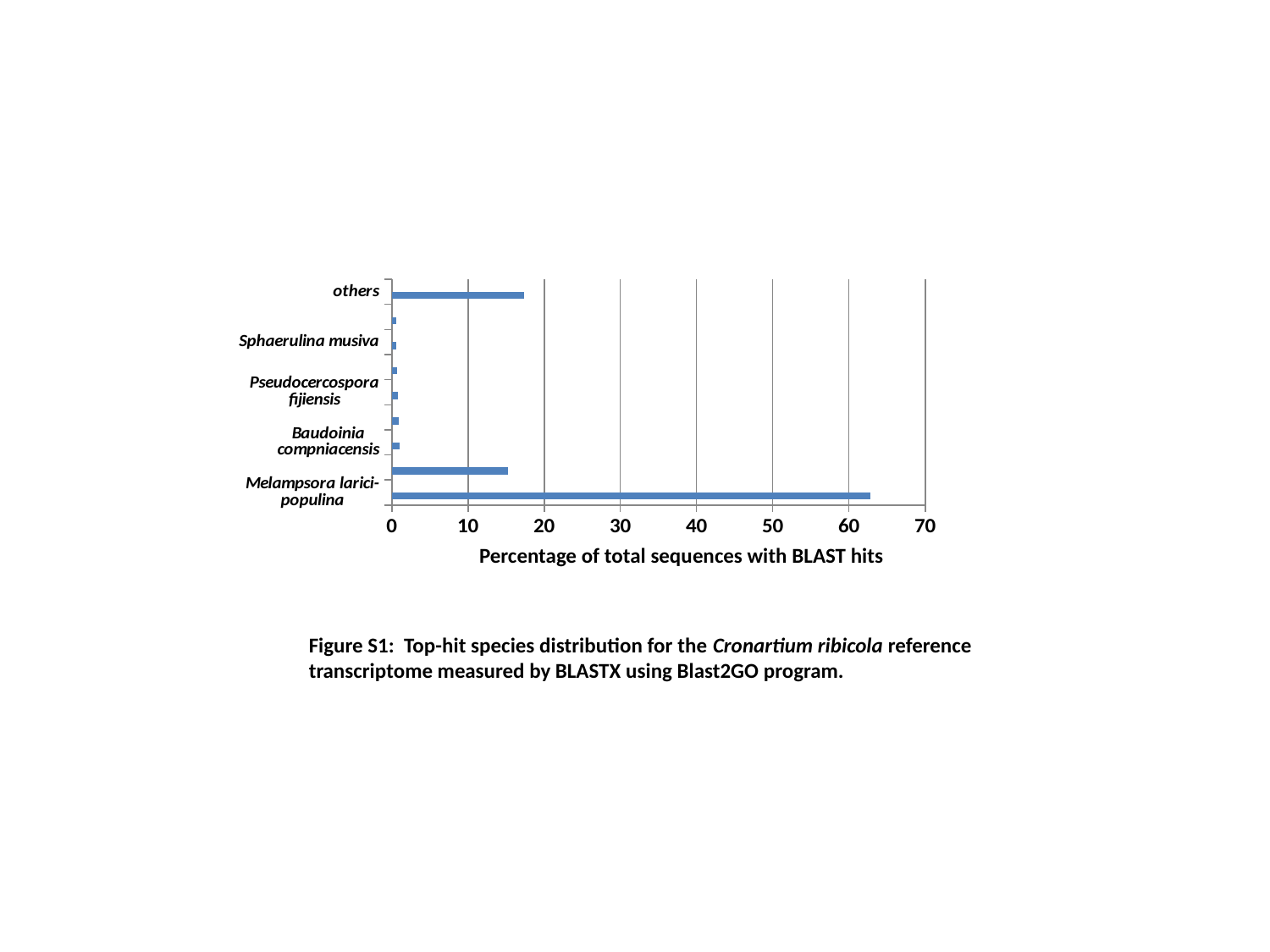

### Chart
| Category | | |
|---|---|---|
| Melampsora larici-populina | 62.81053717071341 | None |
| Puccinia graminis | 15.205774819536892 | None |
| Baudoinia compniacensis | 1.0124683603637386 | None |
| Dothistroma septosporum | 0.9280959970000937 | None |
| Pseudocercospora fijiensis | 0.759351270272804 | None |
| Zymoseptoria tritici | 0.7406018561919939 | None |
| Sphaerulina musiva | 0.6281053717071341 | None |
| Coniosporium apollinis | 0.5812318365051092 | None |
| others | 17.333833317708823 | None |Percentage of total sequences with BLAST hits
Figure S1: Top-hit species distribution for the Cronartium ribicola reference transcriptome measured by BLASTX using Blast2GO program.
