# Supplementary material for: Transcriptome analysis of the white pine blister rust pathogen Cronartium ribicola: de novo assembly, expression profiling, and identification of candidate effectors
Source: BMC Genomics. 2015 Sep 4;16(1):678. doi: 10.1186/s12864-015-1861-1 (PMC4559923; doi:10.1186/s12864-015-1861-1)
Supplement: Additional file 3: Figure S2. — Functional classification of the Cronartium ribicola reference transcriptome assembled de novo from RNA-seq data based on gene ontology (GO). Subcategories of biological process, molecular function, and cellular component are indicated as: GO:0008152, metabolic process; GO:0009987, cellular process; GO:0044699, single-organism process; GO:0050896, response to stimulus; GO:0051179, localization; GO:0065007, biological regulation; GO:0071840, cellular component organization or biogenesis; GO:0032502, developmental process; GO:0023052, signaling; GO:0032501, multicellular organismal process; GO:0000003, reproduction; GO:0040007, growth; GO:0051704, multi-organism process; GO:0040011, locomotion; GO:0002376, immune system process; GO:0022610, biological adhesion; GO:0048511, rhythmic process; GO:0001906, cell killing; GO:0005488, binding; GO:0003824, catalytic activity; GO:0005198, structural molecule activity; GO:0005215, transporter activity; GO:0009055, electron carrier activity; GO:0001071, nucleic acid binding transcription factor activity; GO:0016209, antioxidant activity; GO:0030234, enzyme regulator activity; GO:0060089, molecular transducer activity; GO:0000988, protein binding transcription factor activity; GO:0004872, receptor activity; GO:0045735, nutrient reservoir activity; GO:0016530, metallochaperone activity; GO:0005623, cell; GO:0043226, organelle; GO:0032991, macromolecular complex; GO:0016020, membrane; GO:0031974, membrane-enclosed lumen; GO:0005576, extracellular region; GO:0009295, nucleoid; GO:0030054, cell junction; GO:0045202, synapse; GO:0031012, extracellular matrix; GO:0055044, symplast. (PPTX 104 kb) [file 12864_2015_1861_MOESM3_ESM.pptx]

## Slide 1
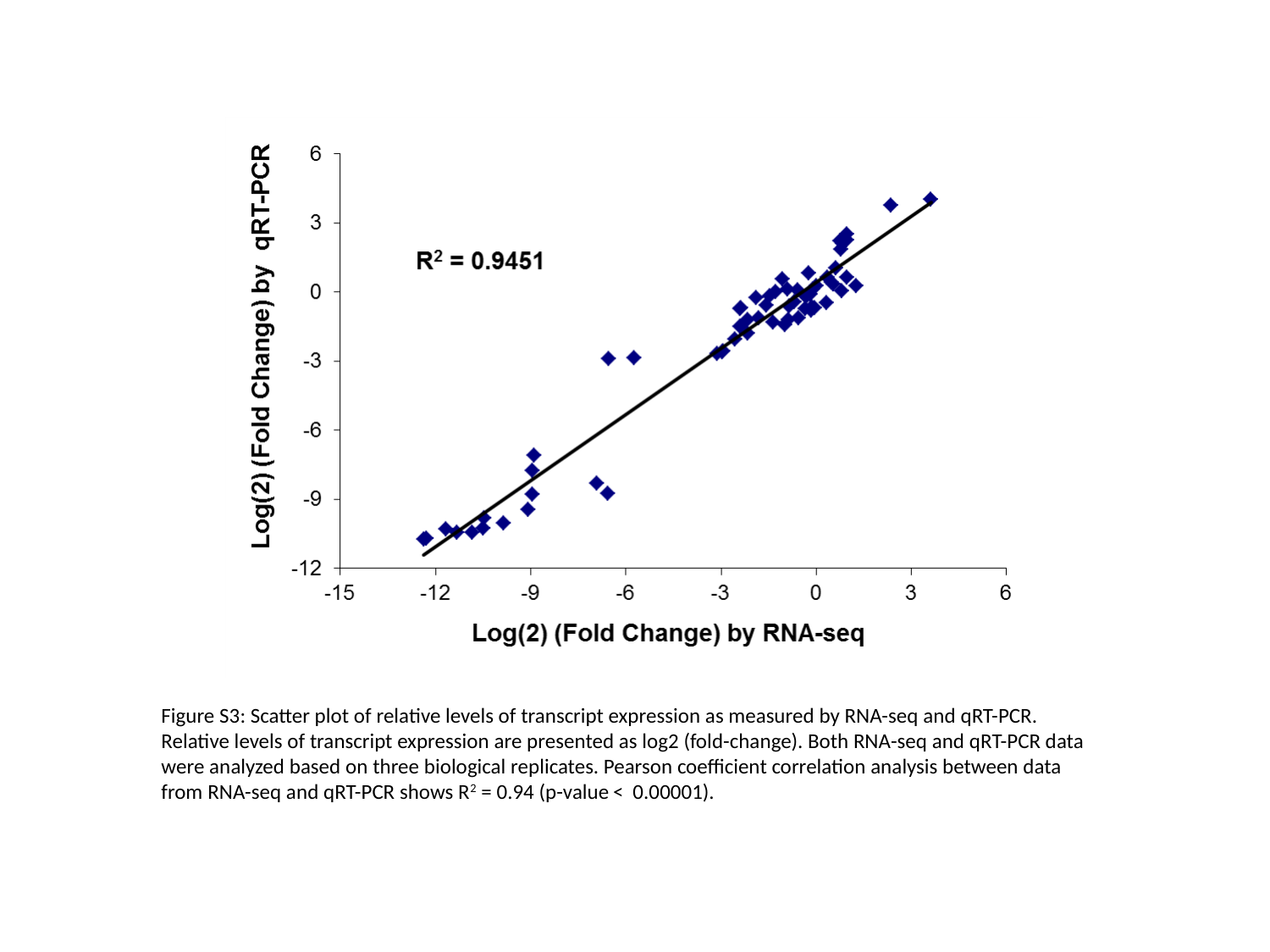

### Chart
| Category |
|---|Figure S3: Scatter plot of relative levels of transcript expression as measured by RNA-seq and qRT-PCR. Relative levels of transcript expression are presented as log2 (fold-change). Both RNA-seq and qRT-PCR data were analyzed based on three biological replicates. Pearson coefficient correlation analysis between data from RNA-seq and qRT-PCR shows R2 = 0.94 (p-value <  0.00001).
